# Silencing of long non-coding RNA HCP5 inhibits proliferation, invasion, migration, and promotes apoptosis via regulation of miR-299-3p/SMAD5 axis in gastric cancer cells

**DOI:** 10.1080/21655979.2020.1863619

**Published:** 2020-12-29

**Authors:** Derong Yin, Xiaohong Lu

**Affiliations:** a Department of Gastroenterology, Hanzhong People’s Hospital, Hanzhong, Shaanxi, PR China; b Department of Gastroenterology, Hubei Key Laboratory of Digestive System Disease, Renmin Hospital of Wuhan University, Wuhan, Hubei, PR China

**Keywords:** Gastric cancer, lncRNA HCP5, microRNA-299-3p, SMAD5

## Abstract

Gastric cancer (GC) is a common malignant gastrointestinal tumor with high mortality. Previous study has reported that the overexpression of lncRNA HCP5 was observed in gastric cancer tissues. The purpose of this study was to investigate the molecular mechanism underlying the effect of lncRNA HCP5 on the proliferative, migratory, and invasive abilities of GC cells. The relative mRNA expression of HCP5, miR-299-3p, and SMAD5 were determined by RT-qPCR. The expressions of proteins associated with apoptosis and invasion were detected by western blot. The interaction of HCP5 with miR-299-3p and SMAD5 with miR-299-3p was confirmed by luciferase reporter assay. The cellular behaviors of AGS cells were, respectively, detected by CCK-8 assays, colony formation assays, migration and invasion assays, and flow cytometry. In our study, lncRNA HCP5 was highly expressed in GC cell lines compared with normal gastric epithelial cell. LncRNA HCP5 silencing inhibited AGS cells proliferation, migration, and invasion, while promoted cell apoptosis. Moreover, miR-299-3p downregulation could abolish the effect of HCP5 knockdown on cellular behaviors of AGS cells. Interestingly, SMAD5 is identified as the downstream target of miR-299-3p, and its expression was inhibited by miR-299-3p. More importantly, SMAD5 silencing inhibited proliferation, migration, and invasion of GC cells, and promoted cell apoptosis. In a word, lncRNA HCP5 silencing inhibits GC cell proliferation, invasion, and migration while promoting its apoptosis via regulation of miR-299-3p/SMAD5 axis. Hence, lncRNA HCP5 could be a novel and promising target for GC treatment.

## Graphical abstract

**Figure:**
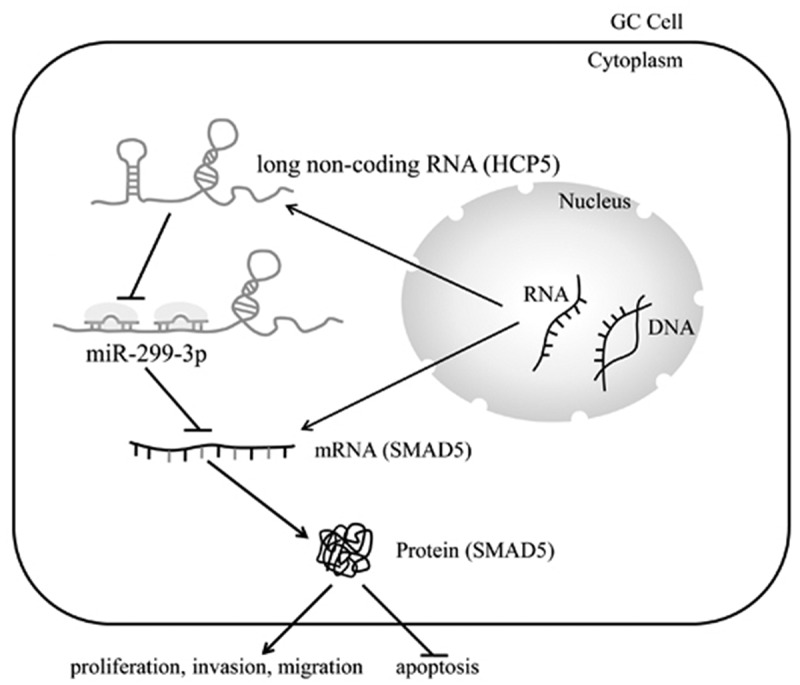

## 1. Introduction

Gastric cancer (GC) is a common malignant gastrointestinal tumor with high mortality, accounting for a huge amount of cancer-related deaths worldwide [1,2]. GC is recognized as an important health challenge because people are at high risk of developing GC, particularly the elderly [3]. Regardless of the development of medical interventions for GC, patients in China still have to face a low 5-year-survival rate of this cancer, particularly at the advanced stages [4,5]. The tumorigenesis of GC is a complicated process, involving complex molecular signaling [6]. Hence, the specific mechanism underlying GC pathogenesis is largely unknown. Therefore, it is desirable to explore the molecular mechanism involving the development and progression of GC.

Long non-coding RNA (lncRNA) is known as a type of endogenous non-coding RNA longer than over 200 nucleotides [7]. LncRNAs have regulative effect on gene expression through participation in physiological processes, including nuclear transportation, alternative splicing, and epigenetics [8]. Research has demonstrated that the level of LncRNA HLA complex P5 (HCP5) was negatively associated with the survival rate of cervical cancer patients. Moreover, lncRNA HCP5 overexpression promoted the progression of cervical cancer via MACC1 overexpression through microRNA-15a adsorption [9]. LncRNA HCP5 was also found to be higher in triple-negative breast cancer tissues and cell lines, and silencing HCP5 led to suppression of cell proliferation and induction of cell apoptosis [10]. Additionally, HCP5 downregulation induced apoptosis and suppressed the proliferative capability of prostate cancer cells [11]. Moreover, a previous study reported that lncRNA HCP5 expression was significantly upregulated in GC tissues compared with those in paired non-tumor tissues [12]. However, the role of lncRNA HCP5 in proliferation, invasion, and migration of GC cells remains undefined. The aim of the present study was to investigate the underlying mechanism implicated the role of lncRNA HCP5 in cellular behaviors of GC cells.

In our study, AGS cell line was used to investigate the relationship between HCP5 and GC. The suppressive effect of HCP5 silencing on cell proliferation, invasion, and migration can be observed, suggesting lncRNA HCP5 may present as an effective target for GC treatment.

## 2. Materials and methods

### 2.1. Cell culture and transfection

Five GC cell lines including NCI-N87, AGS, SNU-1, SNU-16, and HGC-27 and one normal gastric epithelial cell line GES-1 were purchased from American Type Culture Collection (ATCC, Manassas, VA, USA). These cell lines were cultured in DMEM (Gibco, MD, USA) containing 10% FBS (Gibco, MD, USA) and 100 U/ml penicillin/streptomycin in a humidified incubator containing 5% CO_2_ at 37°C.

Small interfering (si) RNA against HCP5 (siRNA-HCP5) or SMAD5 (siRNA-SMAD5), corresponding control siRNA, miR-299-3p inhibitors, and inhibitor-NC were from GenePharma (Shanghai, China). The si-HCP5 (100 nM), si-SMAD5 (100 nM) or negative controls (si-NC), miR-299-3p inhibitors (1 μM) and inhibitor-NC were transfected into the AGS cells using lipofectamine 3000 reagent (Invitrogen, Carlsbad, CA) according to the manufacturer’s instructions. After 48 h of incubation, the transfected AGS cells were used for further experiments and qRT-PCR was performed to confirm transfection efficiency.

### 2.2. RT-qPCR

Total RNA was extracted in transfected cells using TRIzol Reagent (Life Technologies). The mRNAs were reversely transcribed to cDNAs using PrimeScript RT reagent kit (TaKaRa, Japan), followed by the qRT-PCR reactions according to the instructions of SYBR® Green Realtime PCR Master Mix (Toyobo, Japan). The 2^­ΔΔCt^ method was applied to calculate the relative mRNA levels of target genes and house-keeping genes.

### 2.3. Cell viability assessment

After transfection, cell viability assay was performed to detect the cell viability. After incubation for 0 h, 24 h, 48 h, and 72 h, cell counting kit-8 (CCK-8) solution (Dojindo, Kumamoto, Japan) was added (10 µl/well) and maintained with AGS cells for another 2 h. The absorbance at 450 nm was measured with a microplate reader (Bio-Rad).

### 2.4. Colony formation assay

After transfection, AGS cells were planted in 6-well plates. The cells were cultured for 2 weeks at 37°C without disturbance during the period of incubation. Afterward, cell colonies were fixed with 4% paraformaldehyde and stained with 0.1% crystal violet solution before being photographed and counted.

### 2.5. Migration assay

The cell migration rate was evaluated by the wound-healing assay. AGS cells were seeded in a six-well plate and cultured with DMEM for 24 h. A straight linear wound was created by scraping the monolayers with the sterile pipette tips, and the floating cells were removed by washing with PBS. Wound closure was captured by a microscope (Nikon) photographing five random fields. The recovered wound area (%) at 24 h was calculated according to the following formula: [(wound width at 0 h) – (wound width at 24 h)]/wound width at 0 h.

### 2.6. Invasion assay

The ability of cell invasion was determined by Transwell chamber assay. In brief, cells (2 × 10^4^ cells/well) were suspended in DMEM containing 1% FBS and added to the upper chamber, matrigel mix was coated at the underside of the upper chamber. Culture medium (500 μl) supplemented with 10% FBS was added to lower chambers to stimulate invasion. The cells remaining on the upper surface were gently removed after 24 h of incubation, while the cells at the lower surface of the membrane were stained with 0.5% crystal violet for 15 min. Finally, the cells were captured in randomly selected fields with an inverted microscope.

### 2.7. Western blot

Proteins were extracted from transfected cells by RIPA lysis buffer containing protease inhibitors, and the protein concentration was quantified using the BCA assay kits. Equal amounts of protein were fractionated with corresponding SDS-PAGE and transferred to PVDF membranes. Primary antibodies against the following proteins were used: Bcl-2, Bax, Caspase3, pro-Caspase3, matrix metalloproteinases3 (MMP3), MMP9, and SMAD5. The band density was analyzed by ImageJ software and normalized to GAPDH.

### 2.8. Flow cytometry

AGS cells were planted in 6-well plates (1 × 10^5^cells/well). The Annexin V-FITC/PI Apoptosis Kit (Thermo Fisher Scientific) was employed to evaluate cell apoptosis following the manufacturer’s instructions. Cells were briefly digested with 0.25% trypsin and then resuspend with 1× Binding Buffer (500 µL). Thereafter, Annexin V-FITC (5 µL) was added and incubated for 20 min, followed by 10 µL PI staining solution. After incubation under darkness for 20 min, the apoptosis rate was analyzed using a flow cytometer.

### 2.9. Luciferase reporter assay

Before transfection, AGS cells were seeded in 24-well plates and cultured at 37°C. The mutant (MUT) sequences or wild type (WT) sequences of HCP5 and SMAD5 in 3ʹ-UTR containing the miR-299-3p binding site were constructed and subcloned into the pGL3 basic plasmid. Thereafter, cells were co-transfected with WT or MUT HCP5 and SMAD5 reporter plasmids and miR-299-3p mimics or its vector using Lipofectamine 3000. After incubation for 48 h, the fireﬂy luciferase activity was detected and normalized to that of fireﬂy and Renilla using the Dual-Luciferase Reporter Assay System (Promega, Madison, WI, USA). All reporter genes and RNA oligonucleotide sequences were all synthesized by GenePharma (Shanghai, China).

### 2.10. Statistical analysis

Statistical data analysis was carried out with GraphPad Prism 5.0. All data were expressed as the means ± standard error (SEM). Differences among the means were analyzed using One-way ANOVA followed by Tukey test. For comparison between two groups, Student’s *t*-test was used. Differences at *P* < 0.05 were considered statistically significant differences.

## 3. Results

### 3.1. LncRNA HCP5 silencing inhibited the proliferation of AGS cells

**Figure 1.**
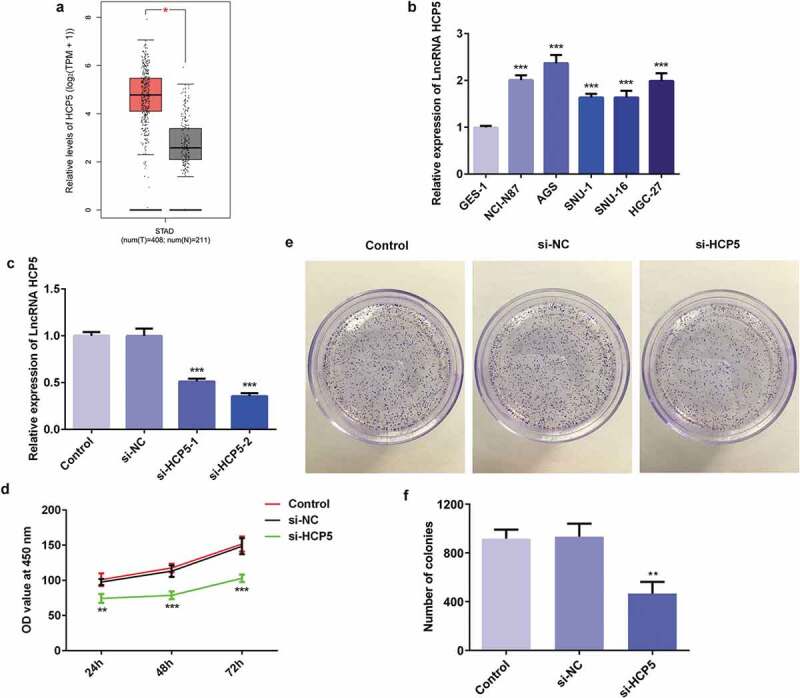
LncRNA HCP5 silencing inhibits the proliferation of AGS cells. (a) The expression level of HCP5 in GC tumor samples and normal samples predicted by GEPIA website. (b,c) The expression of HCP5 was detected by RT-qPCR. (d) The survival rate of cells was evaluated with cell viability assay. (e) The cell proliferation was assessed by colony formation assay, and quantification (f). Error bars represent the mean ± SEM from three independent experiments. **P< 0.01, ***P< 0.001 *vs*. Control

To investigate the association between HCP5 and GC pathophysiology, the level of HCP5 was quantified with RT-qPCR. The HCP5 level in GC patient tumor samples is increased compared to normal samples (Figure 1(a)). Moreover, HCP5 was noticeably upregulated in human GC cell lines including NCI-N87, AGS, SNU-1, SNU-16, and HGC-27 compared with that in normal gastric epithelial cell line GES-1 (Figure 1(b)), suggesting that HCP5 is closely related to GC progression. The AGS cell line, in which HCP5 was expressed at the highest level, was selected for the next experiments. Afterward, siRNA-HCP5 was constructed to downregulate the expression of HCP5. The RT-qPCR results demonstrated that lower expression of HCP5 was detected in siRNA-HCP5-2 group than siRNA-HCP5-1 group (Figure 1(c)). Therefore, siRNA-HCP5-2 was employed for further study.

To investigate the underlying mechanism of HCP5 in the onset and development of GC, cell viability, and colony formation assays were performed. CCK8 results showed that the AGS cells transfected with si-HCP5 had lower survival rate compared with the controls (Figure 1(d)). Meanwhile, the proliferative ability of AGS cells was inhibited by si-HCP5 transfection (Figure 1(e)). These results suggested that lncRNA HCP5 silencing inhibited AGS cells proliferation.

### 3.2. LncRNA HCP5 silencing inhibited the migratory and invasive abilities of AGS cells

**Figure 2.**
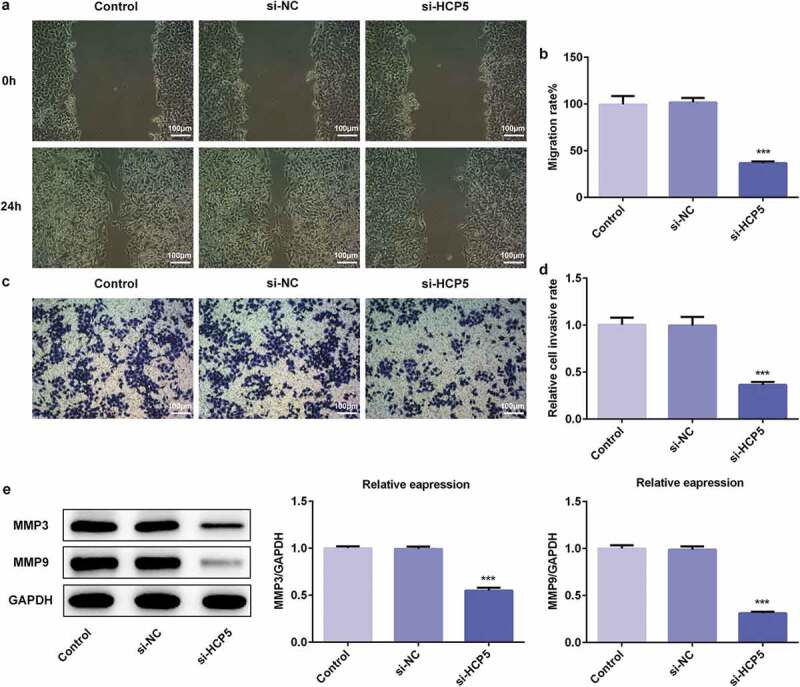
LncRNA HCP5 silencing inhibits the migratory and invasive abilities of AGS cells. (a,b) The cell migration was analyzed by wound-healing assay. Scale bars = 100 μm. (c,d) The cell invasion was determined using transwell assay. Scale bars = 100 μm. (e) The expression levels of MMP3 and MMP9 were determined by western blot. Error bars represent the mean ± SEM from three independent experiments. ***P< 0.001 *vs*. Control

To further investigate the underlying mechanism of HCP5 in the development and progression of GC, the migratory and invasive abilities of AGS cells were measured by migration and invasion assays, respectively. As shown in Figure 2(a,Figure 2b), HCP5 silencing significantly inhibited the migratory ability of AGS cells compared with the controls. Interestingly, HCP5 knockdown also led to reduction in the invasive ability of AGS cells transfected with si-HCP5 compared with the controls (Figure 2(c,Figure 2d)). Thereafter, western blot was performed to quantify the expression levels of matrix metalloproteinases (MMP)-3 and MMP-9, which were involved in tumor invasion and metastasis [13,14]. The western blot results showed that si-HCP5 transfection remarkably suppressed the expression levels of MMP-3 and MMP-9 compared with the controls (Figure 2(e)). These results implied that lncRNA HCP5 silencing inhibited the migratory and invasive abilities of AGS cells.

### 3.3. LncRNA HCP5 silencing promoted the apoptosis of AGS cells

**Figure 3.**
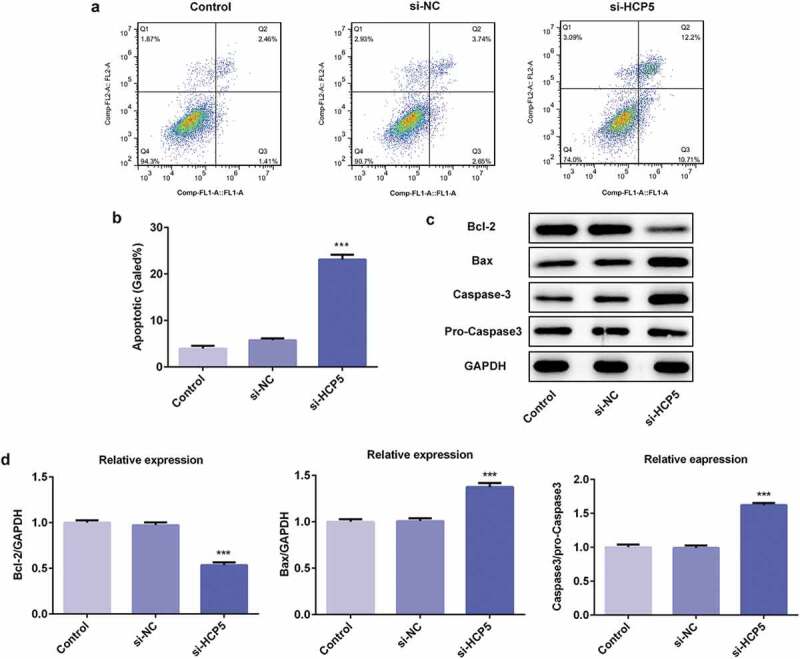
LncRNA HCP5 silencing promotes the apoptosis of AGS cells. (a,b) The cell apoptosis rate was determined by flow cytometry. (c,d) The expression levels of Bcl-2, Bax, Caspase-3 and pro-Caspase-3 were determined by western blot, the GAPDH was set as the internal normalization control. Error bars represent the mean ± SEM from three independent experiments. ***P< 0.001 *vs*. Control

To further study the role of HCP5 in the development and progression of GC, the apoptosis of AGS cells was measured by flow cytometry after Annexin V staining. As shown in Figure 3(a,Figure 3b), AGS cells transfected with si-HCP5 showed higher mortality than the controls. The western blot results demonstrated that lncRNA HCP5 silencing caused reduction of anti-apoptotic proteins (Bcl-2) [15] while upregulation of pro-apoptotic proteins (Bax) [16] and caspase3 (Figure 3(c,Figure 3d)), suggesting HCP5 knockdown promoted the apoptosis of AGS cells.

### 3.4. LncRNA HCP5 silencing led to increase in miR-299-3p expression

**Figure 4.**
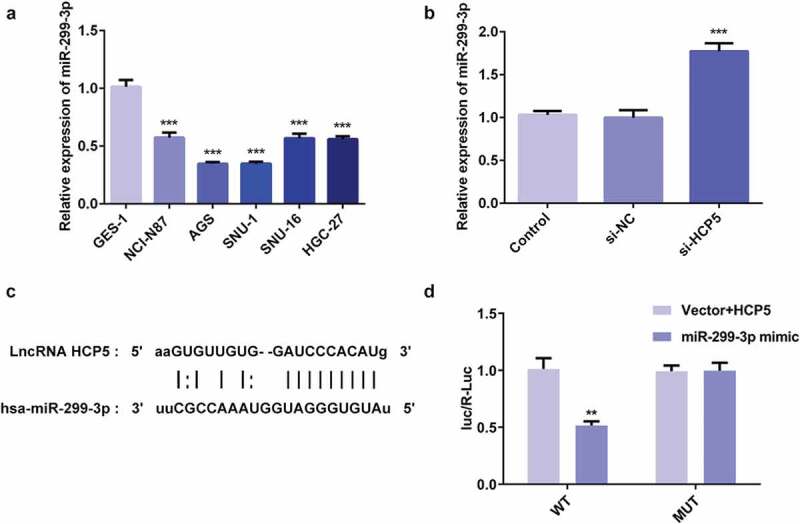
LncRNA HCP5 silencing leads to increase in miR-299-3p expression. (a,b) The relative expression of miR-299-3p was detected by RT-qPCR. (c) 3ʹ-UTR regions of HCP5 is partially complementary to miR-299-3p. (d) The relative luciferase activities in AGS cells transfected with wild-type (WT) or mutated (MUT) HCP5 reporter plasmids and miR-299-3p mimics or vector. Error bars represent the mean ± SEM from three independent experiments. **P< 0.01, ***P< 0.001 *vs*. Control

Firstly, it was observed that the level of miR-299-3p was decreased in human GC cell lines including NCI-N87, AGS, SNU-1, SNU-16, and HGC-27 compared with that in GES-1cells, as demonstrated by RT-qPCR (Figure 4(a)). Then, the upregulation of miR-299-3p was observed under the condition of HCP5 silencing (Figure 4(b)). Interestingly, the interaction between HCP5 and miR-299-3p can be predicted on the Starbase website (http://starbase.sysu.edu.cn/index.php), as presented in Figure 4(c). Afterward, Luciferase reporter assay was performed to confirm whether miR-299-3p is the direct target of HCP5. As shown in Figure 4(d), the relative luciferase activity was significantly lower in AGS cells co-transfected with wild-type luciferase plasmids and miR-299-3p mimic, compared with controls. These results indicated that miR-299-3p specifically binds to the 3ʹUTR of the HCP5, and its expression was reduced by HCP5.

### 1.1. LncRNA HCP5 silencing inhibited the proliferation, invasion, migration while promoted apoptosis via upregulation of miR-299-3p in AGS cells

**Figure 5.**
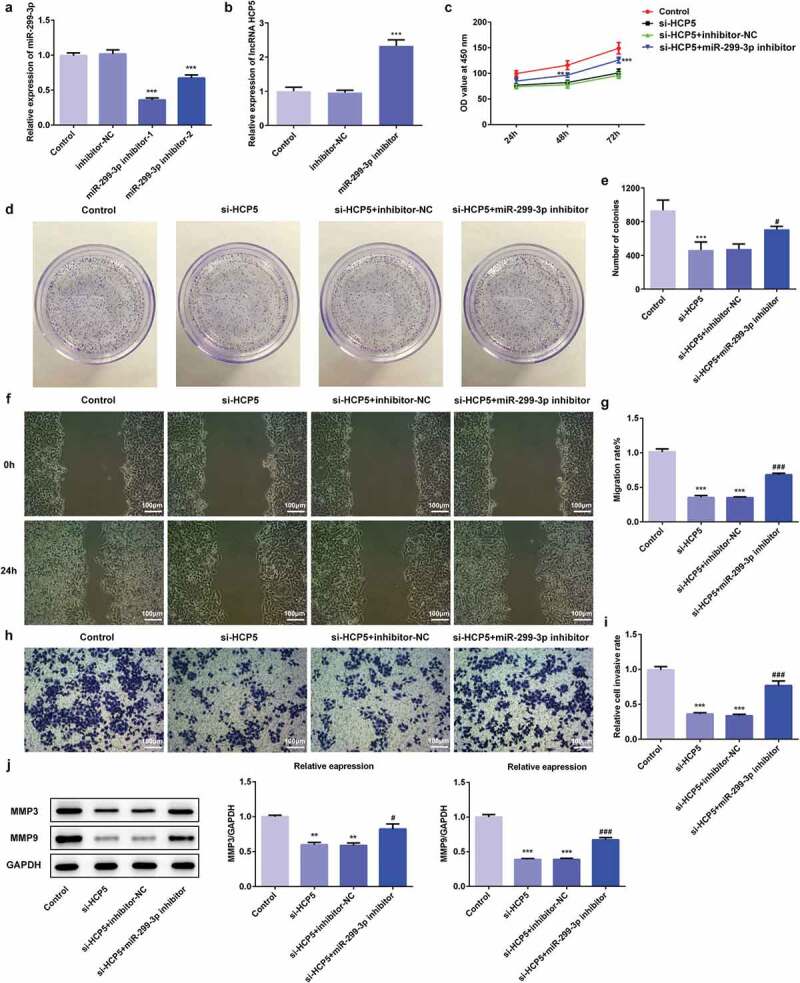
LncRNA HCP5 silencing inhibits proliferation, invasion and migration via upregulation of miR-299-3p in AGS cells. (a) The relative expression of miR-299-3p was detected by RT-qPCR. (b) The relative mRNA level of HCP5 was analyzed by RT-qPCR. (c) The survival rate of cells transfected with or without si-HCP5 and miR-299-3p inhibitor was evaluated with cell viability assay. (d) The cell proliferation was assessed by colony formation assay, and quantification (e). (f,g) The cell migration was analyzed by wound-healing assay. Scale bars = 100 μm. (h,i) The cell invasion was determined using transwell assay. Scale bars = 100 μm. (j) The expression levels of MMP3 and MMP9 were analyzed by western blot. Error bars represent the mean ± SEM from three independent experiments. **P< 0.01, ***P< 0.001 *vs*. Control; ^#^*P*< 0.05, ^###^*P*< 0.001 *vs*. si-HCP5

**Figure 6.**
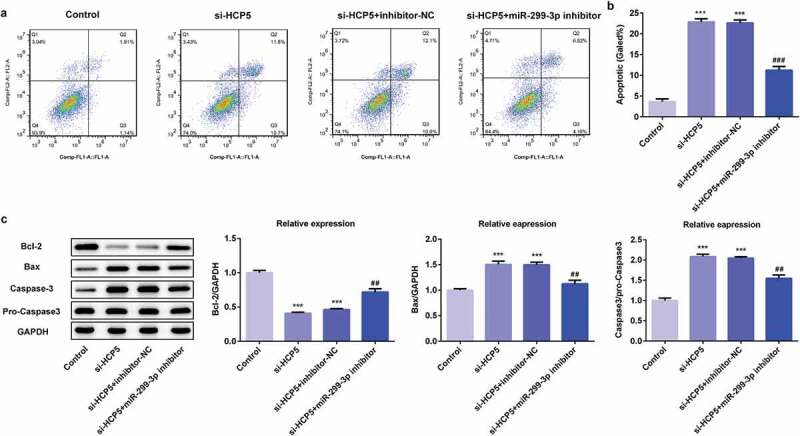
LncRNA HCP5 silencing promotes apoptosis via upregulation of miR-299-3p in AGS cells. (a,b) The cell apoptosis rate was determined by flow cytometry. (c) The expression levels of Bcl-2, Bax, Caspase-3 and pro-Caspase-3 were determined by western blot, the GAPDH was set as the internal normalization control. Error bars represent the mean ± SEM from three independent experiments. ***P< 0.001 *vs*. Control; ^##^*P*< 0.01, ^###^*P*< 0.001 *vs*. si-HCP5

To explore the molecular mechanism related the role of HCP5 in GC progression, the proliferation, invasion, migration, and apoptosis of AGS cells were examined after the combination of HCP5 knockdown and miR-299-3p inhibition in AGS cells. Firstly, the miR-299-3p inhibitor was synthesized to downregulate the miR-299-3p expression. The RT-qPCR showed that miR-299-3p inhibitor-1 was more effective in inhibiting miR-299-3p expression than miR-299-3p inhibitor-2 (Figure 5(a)), therefore miR-299-3p inhibitor-1 was selected for further experiments. As shown in Figure 5(b), miR-299-3p inhibitor-induced HCP5 upregulation. CCK8 and colony formation assays results showed that the miR-299-3p downregulation blocked the inhibitive effect of HCP5 silencing on viability and proliferation of AGS cells (Figure 5(c,Figure 5d)). In addition, migration assay showed that miR-299-3p downregulation reversed the suppressive effect of HCP5 knockdown on the migratory ability of AGS cells (Figure 5(e,Figure 5f)). Moreover, the invasion assay suggested that miR-299-3p downregulation abolished the suppressive effect of HCP5 silencing on invasive ability of AGS cells (Figure 5(g,Figure 5h)), as well as the expression of MMP3 and MMP9 (Figure 5(i)). Finally, flow cytometry results presented that miR-299-3p downregulation reversed the promotive effect of HCP5 knockdown on AGS cells apoptosis (Figure 6(a,Figure 6b)), and western blot results showed that miR-299-3p inhibitor abolished the effect of HCP5 knockdown on the expressions of apoptosis-related proteins (Figure 6(c)). These results indicated that miR-299-3p downregulation accelerated the GC process, and HCP5 silencing inhibits the proliferation, invasion, migration, and promotes the apoptosis via upregulation of miR-299-3p in AGS cells.

### 3.5 SMAD5 expression is increased both in vivo and vitro and regulated by miR-299-3p

**Figure 7.**
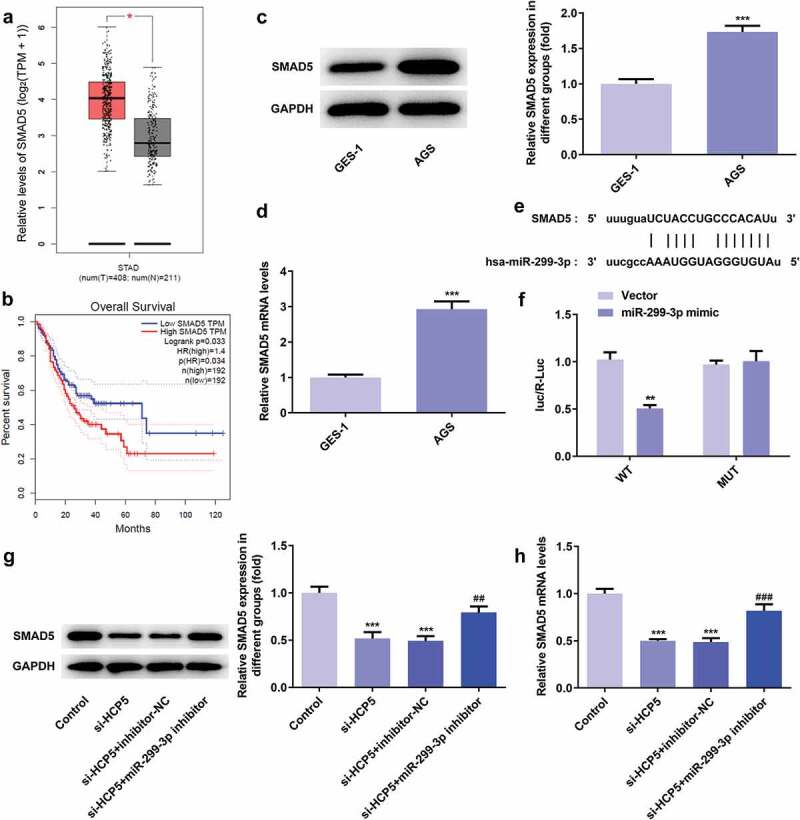
SMAD5 expression is increased both in vivo and vitro and regulated by miR-299-3p. (a) The expression level of SMAD5 in GC tumor samples and normal samples predicted by GEPIA website. (b) The overall survival in STAD patients predicted by GEPIA websites. (c) The proteins expression of SMAD5 was determined by western blot. (d) The mRNA level of SMAD5 was quantified by RT-qPCR. (e) The binding site of has-miR-299-3p on SMAD5. (f) The interaction of SMAD5 with miR-299-3p was confirmed by luciferase reporter assay. (g) The proteins expression of SMAD5 were determined by western blot. (h) The mRNA level of SMAD5 were quantified by RT-qPCR. Error bars represent the mean ± SEM from three independent experiments. **P< 0.01, ***P< 0.001 *vs*. Control; ^##^*P*< 0.01, ^###^*P*< 0.001 *vs*. si-HCP5

To identify the downstream target of miR-299-3p, the interaction between miR-299-3p and SMAD5 were predicted on the Starbase website. The binding site of has-miR-299-3p on SMAD5 was shown in Figure 7(e). Importantly, SMAD5 expression was increased both in GC patient tumor samples compared to normal samples, and its expression level is related to poor survival rate of GC patients on GEPIA website (Figure 7(a,Figure 7b)), suggesting SMAD5 is closely related to GC progression. Moreover, it is detected that the protein and mRNA expression of SMAD5 were upregulated in AGS cells compared to GES-1 cells (Figure C-D). The relative luciferase activity was notably lower in AGS cells co-transfected with wild-type luciferase plasmids and miR-299-3p mimic compared with controls (Figure 7(f)), suggesting that miR-299-3p specifically binds to the 3ʹUTR of the SMAD5. As shown in Figure 7(g,h), the protein and mRNA of SMAD5 were suppressed by HCP5 silencing, and upregulated by miR-299-3p inhibitor. These results suggested that the downstream target of miR-299-3p is SMAD5, which is closely related to GC progression.

### 3.6 SMAD5 silencing inhibited the proliferation, invasion, migration while promoted apoptosis of AGS cells

**Figure 8.**
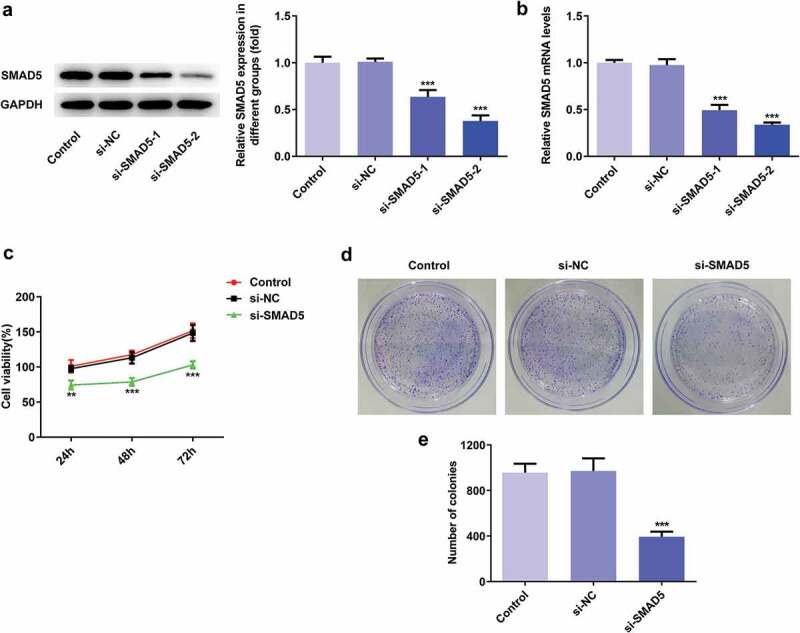
SMAD5 silencing inhibited AGS cell proliferation. (a) The proteins expression of SMAD5 was determined by western blot. (b) The mRNA level of SMAD5 was quantified by RT-qPCR. (c) The viability of AGS cells transfected with or without si-SMAD5 was evaluated with cell viability assay. (d) The cell proliferation was assessed by colony formation assay, and quantification (e). Error bars represent the mean ± SEM from three independent experiments. ***P< 0.001 *vs*. Control

**Figure 9.**
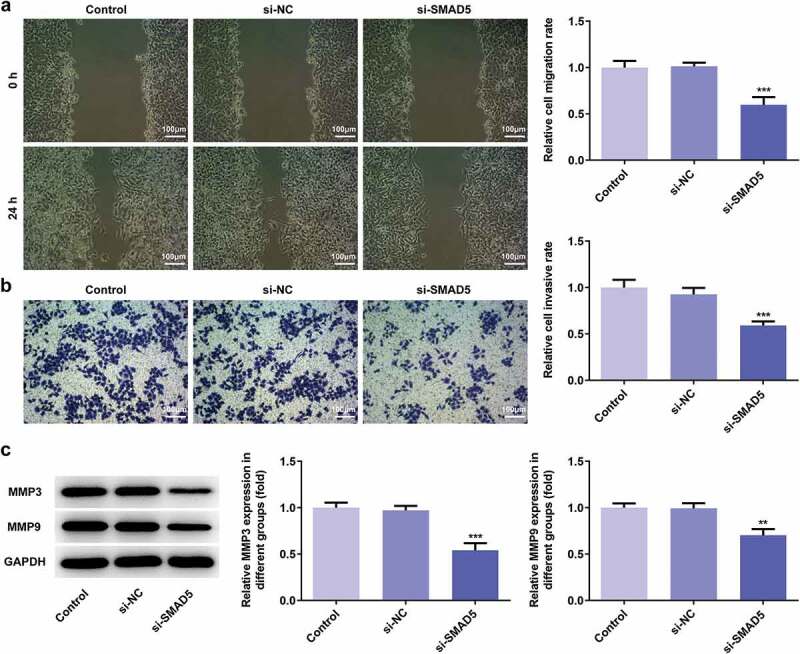
SMAD5 silencing inhibited AGS cells invasion and migration. (a) The cell migration was analyzed by wound-healing assay. Scale bars = 100 μm. (b) The cell invasion was determined by transwell assay. Scale bars = 100 μm. (c) The expression levels of MMP3 and MMP9 were analyzed by western blot. Error bars represent the mean ± SEM from three independent experiments. **P< 0.01, ***P< 0.001 *vs*. Control

**Figure 10.**
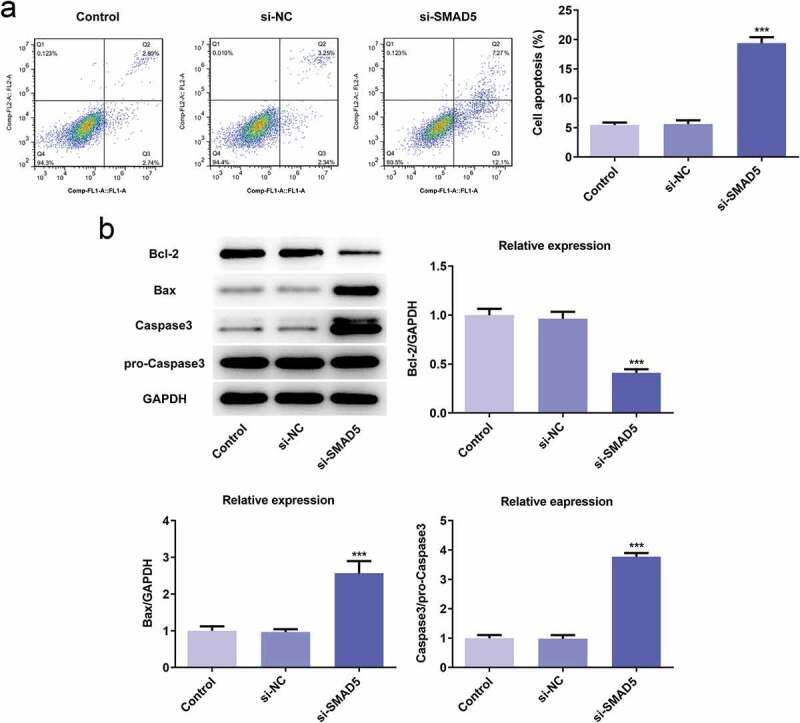
SMAD5 silencing promoted AGS cells apoptosis. (a) The cell apoptosis rate was determined by flow cytometry. (b) The expression levels of Bcl-2, Bax, Caspase-3 and pro-Caspase-3 were determined by western blot, the GAPDH was set as the internal normalization control. Error bars represent the mean ± SEM from three independent experiments. ***P< 0.001 *vs*. Control

To investigate the role of SMAD5 in GC progression, the cellular behaviors of AGS cells were analyzed when SMAD5 expression is downregulated. Firstly, si-SMAD5-1 and si-SMAD5-2 were conducted to achieve SMAD5 silencing. As shown in Figure 8(a,Figure 8b), the protein and mRNA level of SMAD5 were downregulated by transfection with si-SMAD5-1 and si-SMAD5-2. Due to better transfection efficiency, the si-SMAD5-2 was selected in subsequent experiments. The results of cell viability and colony formation assays showed that SMAD5 silencing suppressed the viability and proliferation of AGS cells (Figure 8(c,Figure 8d)). Moreover, the migratory and invasive capabilities were depressed in si-SMAD5 group compared to that in control group (Figure 9(a,Figure 9b)), as well as MMP3 and MMP9 expressions (Figure 9(c)). Additionally, SMAD5 silencing induced higher apoptosis rate of AGS cells compared to controls (Figure 10(a)). The expression levels of Bax and Caspase3 were increased, while Bcl-2 was decreased in si-SMAD5 group compared control group (Figure 10(b)). These results implied that SMAD5 silencing inhibited the proliferation, invasion, migration while promoted apoptosis of AGS cells.

## 4. Discussion

GC is a common malignancy of the digestive system with no remarkable symptoms, which is recognized as one of the primary causes for cancer-related death in the world [17]. Recently, it has been well established that lncRNAs with abnormal expression is closely related to tumorigenesis and cancer progression [18–20], such as gastric cancer, colon cancer, and breast cancer. Accumulating studies have noted that some lncRNAs were differentially expressed in GC tissues compared to adjacent normal tissues. For example, Chen *et al*. identified that lncRNA PCAT18 was poorly expressed in GC tissues and cells [21]. Shi *et al*. reported that the expression of lncRNA CADM1-AS1 was significantly decreased in tumor tissues of GC, and its expression was positively relative to the survival rate and prognosis after clinical treatment [22]. Interestingly, by analysis of microarray detection data, a recent study reported that HCP5 is one of the upregulated lncRNAs in GC tissues [12]. It is also found the HCP5 level in GC patient tumor samples is increased compared to normal samples on GEPIA website. However, the specific mechanism involving the role of HCP5 in GC pathogenesis remains largely unknown. The aim of this study was to investigate the relationship between HCP5 and GC and explore its molecular mechanism in GC progression.

In our study, HCP5 was highly expressed in human GC cell lines compared with that in normal gastric epithelial cells, which was consistent with previous study. Previous study suggested that overexpression of HCP5 promoted the proliferative, migratory, invasive, and angiogenic capabilities of follicular thyroid carcinoma cells [23]. Interestingly, the data indicated that lncRNA HCP5 silencing inhibited the proliferation, migration, and invasion of GC cells. In addition, HCP5 knockdown promoted the apoptosis of AGS cells. Our findings suggested HCP5 may be a tumor regulator in the GC progression.

To further explore the molecular mechanism underlying HCP5 in the development and progression of GC, the putative HCP5 binding sites were predicted on the Starbase website. Interestingly, miR-299-3p was found to be one of the potential targets of HCP5. The interaction between miR-299-3p and HCP5 was confirmed as revealed by luciferase reporter assay data. Moreover, HCP5 downregulation induced significant overexpression of miR-299-3p, suggesting miR-299-3p mediated the role of HCP5 in the tumorigenesis and progression of GC. It was reported that miR-299-3p was notably decreased in thyroid cancer tissues and cells, and miR-299-3p upregulation could suppress cell proliferation and cell cycle progression, while remarkably promote cell apoptosis in thyroid cancer cells [24]. Yu et al. suggested that the expression level of miR-299-3p was downregulated in cervical cancer cell lines, and miR-299-3p inhibited cervical cancer cell growth and invasion [25]. Furthermore, the inhibitory role of miR-299-3p in the migration, invasion, and proliferation of cancer cells was also reported in hepatocellular carcinoma [26]. Importantly, the suppressive effect of miR-299-3p on the viability, migration, and invasion of cancer cells was also confirmed by our results. Our data also demonstrated that miR-299-3p downregulation reversed the suppressive effect of HCP5 knockdown on the proliferation, migration, and invasion of GC cells. In addition, miR-299-3p downregulation abolished the promotive effect of HCP5 silencing on the apoptosis of GC cells. Our finding indicated that lncRNA HCP5 silencing inhibits proliferation, invasion, and migration while promotes the apoptosis via upregulation of miR-299-3p in GC cells.

To identify the downstream target of miR-299-3p, the potential targets of miR-299-3p were predicted by Starbase website. It is found that miR-299-3p can directly target SMAD5 and downregulate its expression. Interestingly, SMAD5 expression was increased both in vivo and vitro, and its expression level is related to poor survival rate of GC patients. Hence, SMAD5 may play an important role in GC progression. SAMD5 mRNA has been reported to be up-regulated in prostate cancer samples, and its high expression is related to poor prognosis after radical prostatectomy [27]. SMAD5 downregulation inhibited cell proliferation, invasion and migration, and reversed EMT, enhanced apoptosis of nasopharyngeal carcinoma cells [28], which is consistent with our study. In our study, SMAD5 silencing inhibited proliferation, migration, and invasion of AGS cells, and enhanced cell apoptosis rate, suggesting SMAD5 was mediated the effect of miR-299-3p in GC progression.

Overall, HCP5 silencing inhibits proliferation, migration, and invasion, and promotes apoptosis via regulation of miR-299-3p/SMAD5 axis in GC cells. Hence, lncRNA HCP5 may presented as an effective and promising target for GC therapies.

## 5. Conclusion

In summary, high HCP5 expression could be an independent risk for the overall survival of GC patients. HCP5 knockdown suppressed the proliferation, migration, and invasion, while promotes the apoptosis of GC cells. HCP5 knockdown induced significant miR-299-3p overexpression and SMAD5 downregulation. Interestingly, miR-299-3p downregulation reversed the effect of HCP5 knockdown on cellular behaviors of GC cells, so as to affect GC progression. As the downstream target of miR-299-3p, SMAD5 silencing inhibited proliferation, migration, and invasion of GC cells, and promoted cell apoptosis. Therefore, HCP5 affected development and progression of GC via regulation of miR-299-3p/SMAD5 axis.

## Supplementary Material

Supplemental Material
