# Supplementary material for: Silencing of long non-coding RNA HCP5 inhibits proliferation, invasion, migration, and promotes apoptosis via regulation of miR-299-3p/SMAD5 axis in gastric cancer cells
Source: Bioengineered. 2020 Dec 29;12(1):225–39. doi: 10.1080/21655979.2020.1863619 (PMC8806318; doi:10.1080/21655979.2020.1863619)
Supplement: Supplemental Material [file KBIE_A_1863619_SM5001.zip › supplement/Highlights.docx]

Highlights

1. LncRNA HCP5 is highly expressed in GC cell lines
2. LncRNA HCP5 silencing inhibited the proliferation and metastatic of AGS cells.
3. MiR-299-3p downregulation abolished the effect of HCP5 knockdown.
