# Supplementary figures and images for: Silencing of long non-coding RNA HCP5 inhibits proliferation, invasion, migration, and promotes apoptosis via regulation of miR-299-3p/SMAD5 axis in gastric cancer cells
Source: Bioengineered. 2020 Dec 29;12(1):225–39. doi: 10.1080/21655979.2020.1863619 (PMC8806318; doi:10.1080/21655979.2020.1863619)

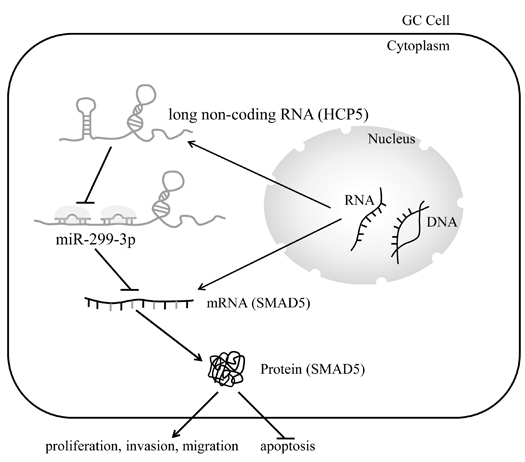

Supplement: Supplemental Material [file KBIE_A_1863619_SM5001.zip › supplement/GraphicalAbstract.jpg]
